# Adherence to Post-Stroke Pharmacotherapy: Scale Validation and Correlates among a Sample of Stroke Survivors

**DOI:** 10.3390/medicina58081109

**Published:** 2022-08-16

**Authors:** Fouad Sakr, Mariam Dabbous, Marwan Akel, Pascale Salameh, Hassan Hosseini

**Affiliations:** 1 UMR 955 INSERM, Institut Mondor de Recherche Biomédicale, Université Paris-Est Créteil, 94010 Créteil, France; 2 École Doctorale Sciences de la Vie et de la Santé, Université Paris-Est Créteil, 94010 Créteil, France; 3 School of Pharmacy, Lebanese International University, Beirut 1105, Lebanon; 4 International Pharmaceutical Federation (FIP), 2517 The Hague, The Netherlands; 5 INSPECT-LB: Institut National de Santé Publique, Épidémiologie Clinique et Toxicologie-Liban, Beirut 1103, Lebanon; 6 School of Medicine, Lebanese American University, Byblos 4504, Lebanon; 7 Faculty of Pharmacy, Lebanese University, Beirut 1103, Lebanon; 8 Department of Primary Care and Population Health, University of Nicosia Medical School, Nicosia 2408, Cyprus; 9 Stroke Unit, Service de Neurologie, CHU Henri Mondor, 94010 Créteil, France

**Keywords:** stroke, post-stroke, stroke survivors, pharmacotherapy, adherence, validated scale, medication, quality of life

## Abstract

*Background and Objectives:* Adherence to post-stroke pharmacotherapy has been less studied compared with other cardiovascular diseases, and previous research in this context utilized generic tools without cross-validating for stroke specific factors and patient characteristics. This study aimed to validate the Lebanese Medication Adherence Scale (LMAS-14) among stroke survivors to assess adherence to post-stroke pharmacotherapy. It also aimed to determine the socioeconomic, clinical characteristics, and health related quality of life correlates of medication adherence among stroke survivors. *Materials and Methods:* This was a cross-sectional study that included stroke survivors from districts throughout Lebanon. A well-structured questionnaire consisting of three parts was developed and utilized to collect data. The first part included questions about the sociodemographic and socioeconomic characteristics. The second part included questions about medical history, current clinical characteristics of the patients, and use of medications. The third part included validated scales to assess stroke outcomes, daily performance and activities, and quality of life. *Results:* A total of 172 stroke survivors were included. The LMAS-14 structure was validated over a solution of three factors, with a Kaiser–Meyer–Olkin (KMO) measure of sampling adequacy = 0.836 and a significant Bartlett’s test of sphericity (*p* < 0.001). Severe difficulty in obtaining medications within the current Lebanese economic crisis was significantly associated with lower medication adherence (Beta = −8.473, *p* = 0.001). Lower medication adherence was also associated with poor stroke prognosis (Beta = −3.264, *p* = 0.027), higher number of used medications (Beta = −0.610, *p* = 0.034), and longer duration of stroke diagnosis (Beta = −4.292, *p* = 0.002). *Conclusions:* The LMAS−14 is a valid and reliable tool to assess medication adherence in stroke practice and research. Severe difficulty in obtaining medications due to unpredictable availability and shortage of supplies is associated with lower medication adherence, and thus places stroke survivors at higher risk of complications and morality. Additional measures and urgent action by stroke care providers and public health stakeholders are necessary to ensure adequate post-stroke management and outcomes.

## 1. Introduction

Stroke mortality has decreased over the past 25 years as a result of medical advancements in stroke therapy, although the severity of stroke-related disabilities has escalated [1]. Stroke survivors frequently need to make significant adaptations to their new health-related quality of life (HRQOL), due to the complications of the disease and the functional limitations it causes [2]. Post-stroke HRQOL manifests complex measures of health that defines physical, mental, and social dimensions among stroke survivors [3]. Previous literature has identified poor post-stroke HRQOL, with physical health and functionality being affected more than other dimensions. In fact, the most reliable predictor of HRQOL is physical disability, though mental health, social support, and comorbidities are also important [4,5,6,7,8,9].

The overall quality of life (QOL) of stroke survivors is also correlated with their adherence to pharmacotherapy for secondary prevention. Adherence to medications is essential for patients to benefit most from the pharmacotherapy in terms of post-stroke morbidity and mortality [10]. Lower rates of recurrent strokes and improved QOL are associated with increased treatment compliance [11]. Nonetheless, poor drug adherence for chronic illnesses was estimated by the World Health Organization (WHO) to be approximately 50% [12]. Adherence is not only a simple measure of compliance with medications; it is a comprehensive evaluation of the reasons for inability or unwillingness to take a prescribed therapy [13]. Healthcare providers should observe the factors that affect medication adherence so that they can provide interventional strategies to achieve concordance in the management plan, which is the state in which both the patient and the healthcare provider are working toward the same intended outcomes [14]. There are significant disparities in health outcomes based on socioeconomic factors, which cannot be entirely explained by standard explanations such as poor health behaviors and practices [15]. Therefore, it is vital to comprehend the socioeconomic, clinical, and HRQOL factors and their correlation with post-stroke medication adherence.

Lebanon is a lower-middle income Arab country located in the Middle East region. Stroke is reportedly the second cause of mortality in the country, and the impact of stroke remains higher compared with developed countries [16,17]. Lebanon is currently facing a severe socioeconomic crisis that ranks among the top three most severe worldwide economic crises. The crisis is associated with a dramatic drop in the value of the local currency estimated to be more than 90%. The economic deterioration has led to shortage of medications and puts the healthcare system at immense risk of public health crisis [18,19]. Urgent interventions are being continuously demanded as cardiovascular complications are becoming more common as a result of the shortages of vital outpatient medications such as antiplatelets, antihypertensives, and antiarrhythmics [19]. Nevertheless, there is a scarcity of data concerning the impact of the economic crisis on the adherence to post-stroke pharmacotherapy.

Many international studies have assessed how well individuals with diabetes mellitus and hypertension adhere to their treatment regimens, yet there has been less research involving post-stroke patients [20,21]. Moreover, previous assessments of post-stroke medication adherence utilized generic scales without cross-validating for stroke specific factors and characteristics among stroke survivors. This study aimed to validate the Lebanese Medication Adherence Scale (LMAS-14) among stroke survivors to assess adherence to pharmacotherapy in stroke care and research. It also aimed to determine the socioeconomic factors, clinical characteristics, and HRQOL correlates of adherence to medications among stroke survivors.

## 2. Materials and Methods

### 2.1. Study Design and Participants

This was a cross-sectional study that included post-stroke patients who survived any episode of stroke. Stroke survivors were defined as patients who ever been hospitalized for an acute cerebrovascular accident and discharged alive regardless of the severity of complications [22]. Patients were recruited from community pharmacies from districts throughout Lebanon. All patients and caregivers presenting to pharmacies to obtain medications that could be prescribed for secondary prevention of stroke or to manage medical conditions that are risk factors for stroke (i.e., hypertension, diabetes mellitus, cardiac arrhythmia, and dyslipidemia) were screened [23]. Patients who were known as stroke patients in the medical profiles of the pharmacies were screened as well. The inclusion criteria were adult patients 18 years of age and above who had ever been diagnosed by a neurologist to have had an ischemic or hemorrhagic stroke. Patients with a history of stroke symptoms without medical diagnosis or prior hospitalization were excluded.

Included patients were interviewed by well-trained healthcare professionals, either face-to-face or through a phone call. The direct caregiver was interviewed on behalf of the patient in cases involving patients with communication disabilities. The interview started with an introduction to explain the objectives of the study and the importance of its outcomes on the QOL of stroke survivors. A well-structured questionnaire in Arabic, the native language of Lebanon, was developed and utilized for data collection that took place between October 2021 and June 2022. The time required to complete the interview and record responses was around 20 min.

### 2.2. Variables and Outcomes

The study questionnaire was divided into three parts. The first part included questions about sociodemographic and socioeconomic characteristics such as age, gender, area of residence, social history, level of education, employment, household income, difficulty in obtaining medications, and health insurance coverage. In this part, financial distress was also assessed by the InCharge Financial Distress/Financial (IFDFW) scale, which is a validated eight-item subjective measure of financial distress and financial well-being. Responses are reported on a scale ranging from 1 to 10 with higher scores indicating better financial well-being [24]. The Cronbach’s alpha of the scale among our sample was 0.936.

The second part of the questionnaire included questions about the past medical history (PMH); stroke parameters relating to the type of stroke, date of diagnosis, and recurrencies; the current clinical characteristics of the patients and use of medications. In this part, the LMAS-14 was used to assess adherence to the prescribed pharmacotherapies. LMAS-14 is a generic 14-item scale that was initially validated among hypertensive patients. It assesses adherence to medications on a 4-grade scale ranging from “always” to “never”. The items of the scale are added to compute the total score, with higher values indicating better adherence to medications [25].

The third part of the questionnaire included validated scales to assess stroke outcomes, daily performance and activities, and QOL. Stroke outcomes were assessed by the modified Rankin Scale (mRS), which assesses the outcomes on a seven-grade scale ranging from the least (no symptoms) to most severe (death) outcome on hospital discharge. The score of the scale is dichotomized to indicate poor prognosis at a cutoff point of 3 and above [26]. The Arabic version of the Stroke Specific Quality of Life (SSQOL-A) scale was used to assess daily performance and post-stroke quality of life. SSQOL-A is a 49-item patient centered scale that assesses energy, upper extremity function, work/productivity, mood, self-care, social roles, family roles, vision, language, thinking, and personality. The score ranges from 49 to 245 with higher scores indicating better QOL [27]. The Cronbach’s alpha of the scale among our sample was 0.979.

The study outcomes were to (1) validate the LMAS-14 among stroke survivors as a tool to assess adherence to prescribed post-stroke pharmacotherapy, (2) determine the impact of the current Lebanese socioeconomic crisis and shortage of supplies on medication adherence among stroke survivors, and (3) determine the clinical and social predictors of post-stroke medication adherence.

### 2.3. Ethical Aspects

The study protocol was approved by the Ethics and Research Committee of the School of Pharmacy at the Lebanese International University (protocol number: 2020RC-048-LIUSOP), who waived the need for a written informed consent as the study was observational and had no clinical interventions. The anonymity and confidentiality of all participants were maintained as personal identifiers were not traced during data collection and analysis.

### 2.4. Sample Size Calculation

The minimal sample size was calculated using CDC’s Epi Info version 7.2.4. for population surveys. The prevalence of stroke in Lebanon is reported to be 3.9% [17]. Accordingly, a minimum sample of 58 stroke survivors is required for post-stroke analyses. For the LMAS-14 validation, a ratio of participants to items should be at least 10:1 [28]. Accordingly, a minimum sample of 140 patients was required for scale validation. Therefore, the minimum sample size required for this study was 140 patients to allow for adequate power of statistical analysis, and produce a 95% confidence level with an acceptable margin of error of 5%.

### 2.5. Statistical Analysis

Data were analyzed using IBM Statistical Package for Social Sciences (IBM SPSS) version 26.0. The sociodemographic, socioeconomic, and clinical characteristics were evaluated by descriptive statistics. Continuous variables were described by their means (±standard deviation, SD), and categorical variables were described by their frequencies and percentages.

Factor analysis with principal component analysis (PCA) was run to validate the LMAS-14 with rotated matrix. The Kaiser–Meyer–Olkin (KMO) measure of sampling adequacy and Bartlett’s test of sphericity were ensured to be adequate. Factors retained in the final scale had Eigen values greater than one. The score of the LMAS-14 was computed from the extracted factors. The correlation of each item of the scale with the whole scale was determined by Pearson correlation. The internal consistency and reliability of the scale were determined by measuring Cronbach’s alpha.

The normal distribution of the score was confirmed by histogram and the Shapiro–Wilk test. The bivariate analysis, taking the LMAS-14 score as the dependent variable, utilized an independent sample T test, one way ANOVA, and Pearson correlation.

A multivariable linear regression was then performed to preclude potential confounding and determine the predictors of adherence to post-stroke pharmacotherapy. Two models were presented taking the LMAS-14 score as the dependent variable, and variables with *p* < 0.2 in the bivariate analysis as independent variables. The first model included the sociodemographic and socioeconomic characteristics, and the second model included the clinical characteristics and stroke parameters. The results are reported as unadjusted Beta with a 95% confidence interval (CI). *p* values lower than 0.05 were considered statistically significant with an acceptable margin of error = 5%.

## 3. Results

### 3.1. Sociodemographic and Socioeconomic Characteristics

A total of 172 stroke survivors were included. The mean age of patients was 62.67 (±13.38), 61.6% were males, 38.4% were ex-smokers, and 70.9% never consumed alcohol. The majority (90.7%) were living within a nuclear or extended family, 70.9% were married, 45.1% had three to four children, and the House Crowding Index mean was 0.90 (±0.52). With respect to employment, 43% were unemployed, 27.9% had a household income between 2,000,000 and 3,500,000 Lebanese Pound (LBP), and the IFDFW scale mean was 39.31 (±19.53). The detailed sociodemographic and socioeconomic characteristics of the patients are shown in Table 1.

### 3.2. Clinical Characteristics and Stroke Parameters

The majority of stroke survivors (83.7%) had a history of one stroke, 66.2% were diagnosed within the last one to five years, and 79.1% of the strokes were ischemic. The mean of number of co-morbidities and used medications were 3.80 (±2.44) and 6.23 (±3.10), respectively. The patients had a mean score of 127.53 (±50.73) for the SSQOL-A, and 76.7% had poor prognosis based on the mRS. The detailed clinical characteristics and stroke parameters of the patients are reported in Table 2. 

### 3.3. Validation of the LMAS-14

#### 3.3.1. Factor Analysis

A factor analysis was performed to validate the LMAS-14 for post-stroke medication adherence. All 14 items of the scale were extracted with Promax rotation. No variables over correlated with each other (r > 0.9), had a low factor loading (<0.3), or low communality (<0.3). The KMO measure of sampling adequacy was 0.836 with a significant Bartlett’s test of sphericity (*p* < 0.001).

The scale converged over a solution of three factors with Eigenvalues greater than 1 and explaining 74.71% of the total variance. The scale’s Factor 1 included seven items representing psychological factors and annoyance, Factor 2 included five items representing forgetfulness, and Factor 3 included two items representing economic factors. Table 3 presents the rotated matrix of the LMAS-14.

#### 3.3.2. Reliability and Internal Consistency

The LMAS-14 had a high Cronbach’s alpha of 0.928 to assess adherence to pharmacotherapy among stroke survivors. The three factors of the scale also had high Cronbach’s alpha values (Table 3). All items of the LMAS-14 significantly correlated with the full scale. The Pearson correlation coefficients ranged from 0.4 to 0.814 with *p* < 0.001. The correlation of the LMAS-14 items with the full scale are shown in Table 4.

### 3.4. Bivariate Analysis of Adherence to Post-Stroke Medications

The LMAS-14 had a mean score of 34.92 (±8.78). The bivariate analysis of the sociodemographic characteristics of the patients showed a significantly higher mean LMAS-14 score for patients who live with family (37.62 ± 3.76) compared with patients who live alone (34.64 ± 9.1). The mean of LMAS-14 score was also significantly different between areas of residence (*p* < 0.001), alcohol consumption status (*p* = 0.03), and number of children (*p* = 0.031). The bivariate associations between LMAS-14 and the socioeconomic characteristics showed significantly different mean LMAS-14 with different levels of difficulty in obtaining medications within the current Lebanese economic crisis and shortage of medications (*p* < 0.001). There was also a significant positive correlation between the scores of LMAS-14 and the IFDFW scale (*p* < 0.001).

The bivariate analysis of clinical characteristics and stroke parameters showed significantly different mean LMAS-14 scores with respect to dates of stroke diagnosis (*p* = 0.027), and significantly lower mean LMAS-14 scores among patients with a poor stroke prognosis on mRS (33.98 ± 9.52) compared with patients with a good prognosis (38 ± 4.57). There was also a significant negative correlation between the number of medications and the LMAS-14 score (*p* = 0.002), and a significant positive correlation between the scores of the SSQOL-A scale and LMAS-14 (*p* = 0.002). The bivariate analyses of the sociodemographic, socioeconomic, stroke parameters, and clinical characteristics of the patients are shown in Table 5 and Table 6.

### 3.5. Predictors of Adherence to Post-Stroke Medications

Two models of multivariable linear regression were performed taking the LMAS-14 as the dependent variable. The first model included the sociodemographic and socioeconomic characteristics as independent variables. A significantly lower score of LMAS-14 was associated with severe difficulty in obtaining medications within the economic crisis and shortage of medications in Lebanon (Beta −8.473, *p* = 0.001). However, a higher LMAS-14 score was significantly associated with living with family (Beta 5.296, *p* = 0.033).

The second model included the clinical characteristics and stroke parameters of the patients as independent variables. A significantly lower LMAS-14 score was associated with being diagnosed with stroke within one to five years (Beta −4.292, *p* = 0.002), an mRS score showing poor prognosis (Beta −3.264, *p* = 0.027), and a higher number of medications taken by patients (Beta −0.610, *p* = 0.034). The multivariable linear regression taking the LMAS-14 score as the dependent variable is reported in Table 7.

## 4. Discussion

The current study validated the LMAS-14 to assess adherence to post-stroke medications. It also determined the socioeconomic, sociodemographic, and clinical predictors of adherence. We found that the extent of adherence is significantly lower within the current Lebanese economic crisis and shortage of drug supplies. We also found a significantly lower adherence among stroke survivors with poor stroke prognosis, history of stroke for more than 1 year, and those who are receiving a higher number of medications. On the other hand, better adherence was significantly associated with living with family.

The LMAS-14 was validated to assess adherence to post-stroke pharmacotherapy. Our findings provide evidence that the scale is a consistent, reliable, and valid method to assess medication adherence among stroke survivors. The scale has three factors related to annoyance (psychological), forgetfulness (cognitive), and economical reasons (socioeconomic factors). The three factors of the scale have a very good internal consistency [29], and the results imply excellent reproducibility because all items correlated highly significantly with the entire scale.

The LMAS-14 was previously validated among hypertensive, diabetic, and hypothyroid patients [25,30,31]. Those studies were able to determine the impact of medication adherence on the extent of control of each medical condition. It was not possible to determine this association in the current study because our sample included only few cases with recurrent stroke. This could be attributed to higher mortality rates among patients with multiple strokes, as previous research reported a 43% higher risk of death after a recurrent stroke [32]. Moreover, higher risks of complications and mortality were already reported as an important outcome of poor adherence to post-stroke pharmacotherapy [33].

The current study found a significantly lower medication adherence as a result of the economic crisis and severe difficulty in obtaining medications due to unavailability and shortage of supplies. A series of political and security incidents in Lebanon have led to a financial crisis, hyperinflation, and depletion of the currency value. The nation’s healthcare system has been severely impacted by this sharp downturn in the economy [34]. Around 80% of the medications used in Lebanon are imported, and the supply of many brands and generic drugs have become erratic and unstable [35]. Our findings showed that the household income of stroke survivors is not significantly associated with lower medication adherence as the multivariable analysis showed no significant association between the two variables. This could imply a higher level of commitment and awareness among stroke survivors to prioritize their needs and budgeting plans in order to maintain financial resources for their therapy, although financial wellbeing (IFDFW scale) had a borderline significant association with medication adherence (*p* = 0.071). Thus, further research involving a larger sample size is recommended to determine this association. The principal impact of the economic downfall on stroke patients appears so far to be related to the shortage of medications and unpredictable drug availability, as our multivariable analysis in this regard only showed a significant negative association between medication adherence and severe difficulty in obtaining medications. There is a scarcity of literature about the impact of economic crises on post-stroke medications adherence, although shortages of cardiovascular medications are a global public health concern as few alternatives are available for many drugs [36]. The present Lebanese economic situation poses major additional challenges on the healthcare system and places stroke survivors at substantial risk of complications and mortality. This necessitates urgent actions by stakeholders to not only prevent these serious outcomes but also minimize the additional economic burden of the subsequent hospitalization and complications.

Poor stroke prognosis characterized by a higher score of mRS was significantly associated with lower medication adherence, suggesting that the functional disabilities after a stroke are associated with lower capability to adhere to prescribed therapies. A previous study has shown no significant association between functional limitations and medication adherence [37]. The major limitation of that study is that it included a relatively small sample size and an unbalanced distribution of the sample over the Lebanese districts. The larger sample size and balanced distribution of stroke survivors in the current study have provided better evidence of the actual association between stroke functional outcomes and medication adherence. Moreover, poor stroke outcomes could be associated with a higher number of prescribed therapies. The current study adds to the literature that a higher number of prescribed post-stroke medications is negatively associated with patient adherence. Our findings are consistent with previous literature that determined lower adherence rates with polypharmacy among patients with chronic conditions [38]. The socioeconomic and cognitive factors of patients could also interact with polypharmacy and predict the medication-adherence behavior. Further research in this context is recommended to determine the influence of stroke mental health outcomes on medication adherence amid socioeconomic difficulties.

Previous literature reported decreased post-stroke medication persistence over time [39]. The current study supports the literature by determining lower adherence to medications after one year of initial stroke diagnosis. Similar findings were also reported by other research that showed a negative correlation between stroke diagnosis duration and self-care including taking medications [40]. This finding suggests that stroke survivors have adaptation difficulties, maybe as a result of poor long-term stroke care. Therefore, there is a need for efficient long-lasting strategies to help stroke survivors manage their chronic condition in a better way. On the other hand, family care and support could be associated with better medication adherence. We found that stroke survivors who live with family adhere more to post-stroke therapy compared with those who live alone. Our results are consistent with other findings that determined better medication adherence as a result of family care and support in the management of other chronic conditions [41,42]. In addition, family support is reportedly associated with better stroke outcomes [43]. Further research on the family function is suggested to identify additional areas of social support that could help stroke survivors who are living alone to adhere better to their medications.

### 4.1. Implications for Practice

Adherence to chronic post-stroke therapy is vital to achieve the intended therapeutic goals and obtain the best long-term outcomes. In general, adherence to long-term therapies is poor [44], and in the setting of stroke it could be influenced by clinical, economical, and social factors. The QOL after a stroke is multidimensional [45], and can correlate with medication adherence for secondary prevention. Therefore, a valid and continuous assessment of adherence to therapy among stroke survivors is warranted due to the complexity of care after a stroke [46]. Our findings show that the LMAS-14 is valid and reliable for use in stroke research and clinical practice. The scale appears useful to assess the extent of adherence to the prescribed post-stroke pharmacotherapy, with lower scores necessitating additional efforts and further patient and caregiver counseling. The economic crisis is complicating post-stroke care and may worsen the prognosis. The unpredictable availability of cerebrovascular and cardiovascular medications mandates urgent action plans by stakeholders to support vulnerable stroke survivors, and reduce the overall impairment of the healthcare system.

### 4.2. Strengths and Limitations

Despite the relatively low prevalence of stroke in Lebanon, this study included a sufficient sample size to allow for adequate power of all statistical analyses that were performed. The sample also included stroke survivors from districts throughout Lebanon, making it possible to assess the impact of the current severe economic crisis while minimizing the risk of selection bias that could be attributed to different socioeconomic characteristics. On the other hand, several limitations couldn’t be avoided. First, the cross-sectional design of the study doesn’t provide temporality, and thus, causality cannot be confirmed. Second, the clinical characteristics and stroke parameters were reported by the patient or/and caregiver, and this could have led to a possible risk of information bias. In addition, participants were recruited from community pharmacies where patients are presumably going to obtain or attempt to obtain medications. Thus, the study did not capture patients who decide not to take medications, or those who have difficulties having their prescriptions delivered to the pharmacy. Prospective research to follow-up hospitalized stroke patients is still recommended to obtain less biased data, although it is believed that such prospective research may still carry a risk of clinical information bias due to possible positive or negative long-term clinical changes that could occur to the different dimensions of HRQOL and thus influence medication adherence. Moreover, the data collection in the current study involved two different methods of collection (face-to-face and phone calls). This could have been also associated with a possible risk of information bias, although it is believed that this risk is minimized as the data collection process involved reporting declared responses rather than observing patient reactions. Third, the current study did not reveal the sensitivity and specificity of the LMAS-14 in determining better adherence to post-stroke medications because no criterion validity was possible. Further research is suggested in this context to compare stroke survivors to stroke-free patients with risk factors for cerebrovascular disease, in order to confirm the validity measures for LMAS-14 in stroke survivors. Finally, residual confounders related to the mental health of stroke survivors cannot be precluded. Future work will determine the mediator role of the psychological QOL dimension on post-stroke medication adherence.

## 5. Conclusions

The LMAS-14 is a valid and reliable tool to assess adherence to post-stroke pharmacotherapy. The Lebanese economic crisis is reducing adherence of stroke survivors to prescribed medications and, thus, could worsen the long-term outcomes of stroke. Indeed, the current unpredictable availability and shortage of medications are associated with lower therapeutic adherence due to severe difficulties in obtaining medications and, therefore, could place stroke survivors at higher risk of complications and mortality. Furthermore, poor stroke prognosis, longer duration of stroke diagnosis, and a higher number of prescribed medications appear to be inversely associated with medication adherence. On the other hand, family care and support appear to play an important positive role. In consequence, it is necessary for stroke care providers and public health stakeholders to devise additional measures and urgent action plans to ensure adequate post-stroke management and outcomes.

## Figures and Tables

**Table 1:** Sociodemographic and socioeconomic characteristics of the patients.

Variable	Stroke Survivors (*n* = 172)	
	Mean or Frequency	SD or %
**Age**	62.67	13.38
Gender		
Male	106	61.6
Female	66	38.4
BMI *	26.81	3.40
**Area of residence**		
Beirut	78	45.30
Bekaa	12	7.0
Mount Lebanon	40	23.30
North	14	8.10
South	28	16.30
**Smoking status**		
Current smoker	62	36.0
Ex-smoker	66	38.4
Non-smoker	44	25.6
**Alcohol consumption**		
In the past, not anymore	36	20.9
No	122	70.9
Yes, currently	14	8.1
**Marital status**		
Divorced	8	4.7
Married	122	70.9
Single	22	12.8
Widowed	20	11.6
**Number of children (*n* = 142)**		
1 to 2	32	22.5
3 to 4	64	45.1
More than 4	46	32.4
**Level of education**		
Illiterate	48	27.9
School level	80	46.5
University level	44	25.6
**Employment**		
Employed	46	26.7
Retired	52	30.2
Unemployed	74	43.0
**Household income**		
<2,000,000 LBP	40	23.3
2,000,000–3,500,000 LBP	48	27.9
3,500,000–5,000,000 LBP	32	18.6
5,000,000–6,500,000 LBP	22	12.8
6,500,000–8,000,000 LBP	6	3.5
>8,000,000 LBP	24	14.0
**Living:**		
Alone	16	9.3
With family (nuclear or extended)	156	90.7
**House Crowding Index**	0.90	0.52
IFDFW scale *	39.31	19.53
**Difficulty in obtaining medications within economic crisis and shortage of medications in Lebanon**		
No	12	7.0
Yes, mild difficulty	56	32.6
Yes, moderate difficulty	50	29.1
Yes, severe difficulty	54	31.4

* BMI: Body Mass Index; IFDFW scale: InCharge Financial Distress/Financial Well-Being scale.

**Table 2:** Clinical characteristics and stroke parameters of the patients.

Variable	Stroke Survivors (*n* = 172)	
	Mean or Frequency	SD or %
**Date of stroke diagnosis**		
Less than 1 year	34	19.8
1 to 5 years	114	66.2
More than 5 years	24	14.0
**Type of stroke**		
Hemorrhagic	36	20.9
Ischemic	136	79.1
**First or recurrent stroke**		
First	144	83.7
Recurrent	28	16.3
**Number of co-morbidities**	3.80	2.44
**Number of medications**	6.23	3.10
mRS *		
Good prognosis	40	23.3
Poor prognosis	132	76.7
SSQOL-A *	127.53	50.73

* mRS: modified Rankin Scale; SSQOL-A: Stroke Specific Quality of Life Scale.

**Table 3:** Promax rotated matrix of the LMAS-14 among stroke survivors.

LMAS-14 Items	Factor 1	Factor 2	Factor 3	Communalities
Stop taking medication if not feeling better during treatment period	0.915			0.749
Stop taking medication if laboratory tests show improvement during treatment period	0.895			0.788
Stop taking medication if it forbids eating certain food because of possible food-medication interaction	0.745			0.548
Stop some of medications if noticed taking too many medications everyday	0.74			0.622
Stop chronic treatment if bored of it	0.689			0.623
Stop taking medication if a neighbor/relative took a similar prescription and it caused them side effects	0.679			0.762
Stop taking medication in case of side effects	0.567			0.603
Forget to take medication if invited to lunch or dinner		0.93		0.787
Forget to take medication		0.918		0.869
Late when it comes to buying medication packs when they become empty		0.891		0.875
Forget to take medication when busy		0.802		0.830
Late when it comes to buying medication when it’s done		0.778		0.795
Stop taking medication if insurance does not cover it			0.915	0.806
Stop buying medication packs if considered them expensive			0.716	0.805
*Percentage of variance explained*	53.5	11.86	9.34	
*Cronbach’s alpha*	0.898	0.925	0.746	

Factor 1 = Psychological/annoyance; Factor 2 = Forgetfulness; Factor 3 = Economical. Cronbach’s alpha for the LMAS-14 = 0.928. Total percentage of variance explained: 74.71%. Kaiser–Meyer–Olkin (KMO) = 0.836. Bartlett’s test of sphericity: *p* < 0.001.

**Table 4:** Pearson correlation of the LMAS-14 items with the full scale among stroke survivors.

LMAS-14 Items	LMAS-14 Item Number	r *	*p*-Value
Stop taking medication if not feeling better during treatment period	9	0.694	<0.001
Stop taking medication if laboratory tests show improvement during treatment period	8	0.753	<0.001
Stop taking medication if it forbids eating certain food because of possible food-medication interaction	6	0.604	<0.001
Stop some of medications if noticed taking too many medications everyday	10	0.699	<0.001
Stop chronic treatment if bored of it	11	0.731	<0.001
Stop taking medication if a neighbor/relative took a similar prescription and it caused them side effects	7	0.801	<0.001
Stop taking medication in case of side effects	12	0.766	<0.001
Forget to take medication if invited to lunch or dinner	2	0.752	<0.001
Forget to take medication	3	0.814	<0.001
Late when it comes to buying medication packs when they become empty	4	0.796	<0.001
Forget to take medication when busy	1	0.811	<0.001
Late when it comes to buying medication when it’s done	5	0.774	<0.001
Stop taking medication if insurance does not cover it	13	0.400	<0.001
Stop buying medication packs if considered them expensive	14	0.714	<0.001

* Pearson correlation coefficient.

**Table 5:** Bivariate analysis of sociodemographic, socioeconomic, stroke parameters, and clinical characteristics of the patients, taking the LMAS-14 as the dependent variable.

Variable	Mean	SD	*p*-Value
**Gender**			0.785
Male	34.77	8.21	
Female	35.15	9.68	
**Living:**			0.017
Alone	34.64	9.1	
With family (nuclear or extended)	37.62	3.76	
**Area of residence**			<0.001 ^a^
Beirut	37.31	6.54	
Bekaa	35.83	7.73	
Mount Lebanon	30.5	9.33	
North	30	11.11	
South	36.64	9.76	
**Smoking status**			0.254
Current smoker	33.9	8.14	
Ex-smoker	34.67	9.14	
Non-smoker	36.73	9.02	
**Alcohol consumption**			0.030 ^b^
In the past, not anymore	31.78	9.72	
No	36.02	8.01	
Yes, currently	33.43	11.02	
**Marital status**			0.743
Divorced	34.25	7.72	
Married	35.38	8.35	
Single	34.09	11.62	
Widowed	33.3	8.56	
**Number of children**			0.031 ^c^
1 to 2	32.25	9.65	
3 to 4	34.72	7.57	
More than 4	37.22	7.76	
**Level of education**			0.083
Illiterate	34.96	9.48	
School level	33.6	8.55	
University level	37.27	8.06	
**Employment**			0.400
Employed	35.48	7.99	
Retired	33.54	8.68	
Unemployed	35.54	9.3	
**Household income**			0.055
<2,000,000 LBP	33.5	10.07	
2,000,000–3,500,000 LBP	32.67	9.26	
3,500,000–5,000,000 LBP	37.5	6.44	
5,000,000–6,500,000 LBP	35.82	8.57	
6,500,000–8,000,000 LBP	32.67	12.21	
>8,000,000 LBP	38.08	5.89	
**Medical coverage/insurance**			0.454
No	33.96	10.09	
Yes, NSSF *	34.74	8.06	
Yes, private medical insurance or private mutual fund (with or without NSSF)	36.76	8.25	
Yes, coverage through the public or military sector (other than NSSF)	34.29	8.31	
**Date of stroke diagnosis**			0.027 ^d^
Less than 1 year	38.24	6.9	
1 to 5 years	33.74	9.28	
More than 5 years	35.83	7.61	
**Type of stroke**			0.814
Hemorrhagic	34.61	9.72	
Ischemic	35	8.55	
**First or recurrent stroke**			0.429
First	35.15	8.88	
Recurrent	33.71	8.28	
**Medications coverage by third party payers**			0.614
No	35.59	9.21	
Yes completely	34.8	8.85	
Yes partially	34.17	8.29	
**Difficulty in obtaining medications within economic crisis and shortage of medications in Lebanon**			<0.001 ^e^
No	39.5	2.07	
Yes, mild difficulty	38.21	6.55	
Yes, moderate difficulty	34.6	8.53	
Yes, severe difficulty	30.78	10.11	
**Obtaining medications from outside the country due to shortage of medications in Lebanon**			0.116
No	33.79	10.13	
Yes, sometime	35.78	7.87	
Yes, always	41	1.85	
Yes, most of the time	34.18	6.5	
mRS *			<0.001
Good prognosis	38	4.57	
Poor prognosis	33.98	9.52	

* NSSF: National Social Security Fund; mRS: modified Rankin Scale. ^a^ Post hoc analysis showed significant difference between “Beirut” and “Mount Lebanon” (*p* < 0.001); and “Beirut” and “North” (*p* = 0.028). ^b^ Post hoc analysis showed significant difference between “No” and “In the past, not any more” (*p* = 0.032). ^c^ Post hoc analysis showed significant difference between “1 to 2” and “More than 4” (*p* = 0.027). ^d^ Post hoc analysis showed significant difference between “Less than 1 year” and “1 to 5 years” (*p* = 0.025). ^e^ Post hoc analysis showed significant difference between “No” and “Yes, severe difficulty” (*p* = 0.007).

**Table 6:** Bivariate analysis of sociodemographic, socioeconomic, and clinical characteristics of the patients, taking the LMAS-14 as the dependent variable.

Variable	Correlation Coefficient **	*p*-Value
Age	0.021	0.785
BMI *	0.131	0.087
House Crowding Index	−0.113	0.141
Number of co-morbidities	−0.125	0.101
Number of medications	−0.235	0.002
SSQOL-A scale *	0.238	0.002
IFDFW scale *	0.307	<0.001

* BMI: Body Mass Index; SSQOL-A scale: Stroke Specific Quality of Life scale; IFDFW Scale: InCharge Financial Distress/Financial Well-Being scale. ** Pearson correlation coefficient.

**Table 7:** Multivariable linear regression taking the LMAS-14 score as the dependent variable.

Model 1 Including Sociodemographic and Socioeconomic Characteristics *					
**Variable**	**Unstandardized Beta**	**Standardized Beta**	***p*-Value**	**95% CI**	
				**Lower**	**Upper**
Living with family versus living alone	5.296	0.179	0.033	0.429	10.163
Difficulty in obtaining medications within economic crisis and shortage of medications in Lebanon: Severe difficulty versus No difficulty	−8.473	−0.264	0.001	−13.404	−3.542
IFDFW scale ^‡^	0.056	0.135	0.071	−0.005	0.117
Model 2 Including Clinical Characteristics and Stroke Parameters **					
**Variable**	**Unstandardized Beta**	**Standardized Beta**	***p*** **-Value**	**95% CI**	
				**Lower**	**Upper**
Date of stroke diagnosis: 1 to 5 years versus less than 1 year	−4.292	−0.252	0.002	−6.937	−1.647
mRS ^‡^: Poor versus Good prognosis	−3.264	−0.172	0.027	−6.152	−0.376
Number of medications	−0.610	−0.171	0.034	−1.175	−0.045

* Variables initially included in the model: area of residence; number of children; level of education; alcohol consumption; household income; obtaining medications from outside the country due to shortage of medications in Lebanon; House Crowding Index. ** Variables initially included in the model: Body Mass Index (BMI); stroke specific quality of life scale (SSQOL-A); number of co-morbidities. ^‡^ IFDFW scale: InCharge Financial Distress/Financial Well-Being scale; mRS: modified Rankin Scale.

## Data Availability

The data presented in this study are available from the corresponding author on reasonable request.

## References

[1] Langhorne P., Sandercock P., Prasad K. (2009). Evidence-based practice for stroke. Lancet Neurol..

[2] Kwok T., Lo R.S., Wong E., Wai-Kwong T., Mok V., Kai-Sing W. (2006). Quality of life of stroke survivors: A 1-year follow-up study. Arch Phys. Med. Rehabil..

[3] Carod-Artal F.J. (2012). Determining quality of life in stroke survivors. Expert Rev. Pharm. Outcomes Res..

[4] King R.B. (1996). Quality of life after stroke. Stroke.

[5] Aström M., Asplund K., Aström T. (1992). Psychosocial function and life satisfaction after stroke. Stroke.

[6] Sturm J.W., Donnan G.A., Dewey H.M., Macdonell R.A., Gilligan A.K., Srikanth V., Thrift A.G. (2004). Quality of life after stroke: The North East Melbourne Stroke Incidence Study (NEMESIS). Stroke.

[7] Carod-Artal J., Egido J.A., González J.L., Varela de Seijas E. (2000). Quality of life among stroke survivors evaluated 1 year after stroke: Experience of a stroke unit. Stroke.

[8] Mackenzie A.E., Chang A.M. (2002). Predictors of quality of life following stroke. Disabil. Rehabil..

[9] Nichols-Larsen D.S., Clark P.C., Zeringue A., Greenspan A., Blanton S. (2005). Factors influencing stroke survivors’quality of life during subacute recovery. Stroke.

[10] Çevik C., Tekir Ö., Kaya A. (2018). Stroke patients’ quality of life and compliance with the treatment. Acta Med. Mediterr..

[11] Arif H., Aijaz B., Islam M., Aftab U., Kumar S., Shafqat S. (2007). Drug compliance after stroke and myocardial infarction: A comparative study. Neurol. India.

[12] World Health Organization Adherence to Long-Term Therapies: Evidence for Action/[Edited by Eduardo Sabaté]. https://apps.who.int/iris/handle/10665/42682.

[13] Thomson P., Rushworth G.F., Andreis F., Angus N.J., Mohan A.R., Leslie S.J. (2020). Longitudinal study of the relationship between patients’ medication adherence and quality of life outcomes and illness perceptions and beliefs about cardiac rehabilitation. BMC Cardiovasc. Disord.

[14] Horne R. (2006). Compliance, adherence, and concordance: Implications for asthma treatment. Chest.

[15] Liu Q., Wang M., Guo J., Li J., Li C., Qian M. (2011). Effect of socioeconomic status on secondary prevention of stroke. Int. J. Qual Health Care.

[16] Lahoud N., Salameh P., Saleh N., Hosseini H. (2016). Prevalence of Lebanese stroke survivors: A comparative pilot study. J. Epidemiol. Glob. Health.

[17] Jurjus A.R., Tohme R.A., Ephrem G., Hussein I.A., Jurjus R. (2009). Incidence and prevalence of circulatory diseases in Lebanon: A physician’s inquiry. Ethn. Dis..

[18] World Bank Lebanon Economic Monitor, Spring 2021: Lebanon Sinking (to the Top 3). https://elibrary.worldbank.org/doi/abs/10.1596/35626.

[19] Khattar G., Hallit J., El Chamieh C., Bou Sanayeh E. (2022). Cardiovascular drug shortages in Lebanon: A broken heart. Health Econ. Rev..

[20] Lin E.H., Von Korff M., Ciechanowski P., Peterson D., Ludman E.J., Rutter C.M., Oliver M., Young B.A., Gensichen J., McGregor M. (2012). Treatment adjustment and medication adherence for complex patients with diabetes, heart disease, and depression: A randomized controlled trial. Ann. Fam. Med..

[21] Viswanathan M., Golin C.E., Jones C.D., Ashok M., Blalock S.J., Wines R.C., Coker-Schwimmer E.J., Rosen D.L., Sista P., Lohr K.N. (2012). Interventions to improve adherence to self-administered medications for chronic diseases in the United States: A systematic review. Ann. Intern. Med..

[22] World Health Organization ICD-10 Version: 2016. https://icd.who.int/browse10/2016/en#/I60-I69.

[23] Boehme A.K., Esenwa C., Elkind M.S.V. (2017). Stroke Risk Factors, Genetics, and Prevention. Circ. Res..

[24] Prawitz A.D., Garman E.T., Sorhaindo B., O’Neill B., Kim J., Drentea P. (2006). The incharge financial distress/financial well-being scale: Establishing validity and reliability. Fin. Counsel. Plan.

[25] Bou Serhal R., Salameh P., Wakim N., Issa C., Kassem B., Abou Jaoude L., Saleh N. (2018). A New Lebanese Medication Adherence Scale: Validation in Lebanese Hypertensive Adults. Int. J. Hypertens.

[26] Banks J.L., Marotta C.A. (2007). Outcomes validity and reliability of the modified Rankin scale: Implications for stroke clinical trials: A literature review and synthesis. Stroke.

[27] Sallam S.A., Al-Khamis F.A., Muaidi Q.I., Abdulla F.A. (2019). Translation and validation of the stroke specific quality of life scale into Arabic. NeuroRehabilitation.

[28] Boateng G.O., Neilands T.B., Frongillo E.A., Melgar-Quiñonez H.R., Young S.L. (2018). Best Practices for Developing and Validating Scales for Health, Social, and Behavioral Research: A Primer. Front. Public Health.

[29] Tavakol M., Dennick R. (2011). Making sense of Cronbach’s alpha. Int. J. Med. Educ..

[30] Ibrahim L., Ibrahim L., Hallit S., Salameh P., Sacre H., Akel M., Bou Serhal R., Saleh N. (2021). Validation of the Lebanese Medication Adherence Scale among Lebanese diabetic patients. Int. J. Clin. Pharm..

[31] Hallit S., Haddad C., Sacre H., Rahme C., Akel M., Saleh N., Chalhoub C., Salameh P. (2021). Medication adherence among Lebanese adult patients with hypothyroidism: Validation of the Lebanese Medication Adherence Scale and correlates. Clin. Epidemiol. Glob. Health.

[32] Lekoubou A., Nkoke C., Dzudie A., Kengne A.P. (2017). Recurrent Stroke and Early Mortality in an Urban Medical Unit in Cameroon. J. Stroke Cerebrovasc. Dis..

[33] Yeo S.H., Toh M., Lee S.H., Seet R.C.S., Wong L.Y., Yau W.P. (2020). Impact of medication nonadherence on stroke recurrence and mortality in patients after first-ever ischemic stroke: Insights from registry data in Singapore. Pharmacoepidemiol. Drug Saf..

[34] Bizri A.R., Khalil P.B. (2021). A Lebanese physician’s dilemma: Not how, but with what?. Lancet.

[35] Das M. (2021). Lebanon faces critical shortage of drugs. Lancet Oncol..

[36] Reed B.N., Fox E.R., Konig M., Jackevicius C.A., Masoudi F.A., Rabinstein A.A., Page R.L. (2016). The impact of drug shortages on patients with cardiovascular disease: Causes, consequences, and a call to action. Am. Heart J..

[37] Saade S., Kobeissy R., Sandakli S., Malaeb D., Lahoud N., Hallit S., Hosseini H., Salameh P. (2021). Medication adherence for secondary stroke prevention and its barriers among lebanese survivors: A cross-sectional study. Clin. Epidemiol. Glob. Health.

[38] Marcum Z.A., Gellad W.F. (2012). Medication adherence to multidrug regimens. Clin. Geriatr. Med..

[39] Jang D.E., Zuñiga J.A. (2020). Factors associated with medication persistence among ischemic stroke patients: A systematic review. Neurol. Res..

[40] Jang D.E., Shin J.H. (2019). Self-Care Performance of Middle-Aged Stroke Patients in Korea. Clin. Nurs. Res..

[41] Gabr W.M., Shams M.E. (2015). Adherence to medication among outpatient adolescents with epilepsy. Saudi Pharm. J..

[42] Mellins C.A., Brackis-Cott E., Dolezal C., Abrams E.J. (2004). The role of psychosocial and family factors in adherence to antiretroviral treatment in human immunodeficiency virus-infected children. Pediatr. Infect. Dis. J..

[43] Miller K.E. (2001). Can family support improve stroke recovery?. Am. Fam. Physician.

[44] Aliotta S.L., Vlasnik J.J., Delor B. (2004). Enhancing adherence to long-term medical therapy: A new approach to assessing and treating patients. Adv. Ther..

[45] Sahu R.K., Kumar S., Yadav P. (2021). A study on the quality of life among stroke survivors: A cross sectional study. J. Med. Res. Innov..

[46] Nelson M.L.A., Hanna E., Hall S., Calvert M. (2016). What makes stroke rehabilitation patients complex? Clinician perspectives and the role of discharge pressure. J. Comorb..

